# Buffalo Academy of Medicine

**Published:** 1902-09

**Authors:** F. W. McGuire


### SOCIETY PROCEEDINGS.

### Buffalo Academy of Medicine.

Reported by F. W. McGUIRE, M. D., Secretary.

ANNUAL meeting held at the academy rooms, Public Library Building, Tuesday, June 17, 1902.

The president, Dr. Charles G. Stockton, called the meeting to order at 8.45 p.m.

The minutes of the last annual meeting were read and approved.

The secretary of Academy and council presented the following report for the session of 1901-1902, which was on motion received and ordered to be spread on the minutes.

ANNUAL REPORT OF SECRETARY.

During the summer a program was prepared for the entire season and a copy of same sent to each member of the Academy.

The past few years the attendance has gradually decreased, and this year a special effort was made to have papers read by men of note from various other cities. The plan, in a measure, has been successful. The average attendance at stated meetings was increased from 39 to 44. The average attendance of various sections: Medical from 36 to 41; obstetrics, 27 to 34; ophthalmology, decreased from 26 to 13; surgery, from 20 to 27; pathology, from 24 to 46.

During the past year we have added more new members to the Academy than has been done on any previous year, due principally to the effort of Drs. Stockton and Wende. The number of new members being 31, and there has been only 6 members dropped for nonpayment of dues. While there has been an increase in the attendance this year over last, it is still below what I think we ought to expect.

There is another method, I believe, by which we can still further increase our attendance. We have in Buffalo some 700 physicians who are eligible for membership in the Buffalo Academy of Medicine, and of these only 200 are members. Now, if we can double our membership I think it fare to assume that our attendance will be correspondingly increased, providing, of course, we keep up the high standing of our meetings.

There has been five stated meetings. In September, section of medicine—Intestinal Dystrypsia, J. C. Hemmeter; Treatment of pneumonia, De Lancey Rochester. Attendance, 30.

In November, section of Ophthalmology—The operation for extraction of cataract, as originally devised by Daviel, A. A. Hubbell. Attendance, 32.

In January, section of Obstetrics and Gynecology—In pelvic suppuration when shall we operate by the abdomen and when by the vagina? William R. Pryor, New York City. Attendance, 58.

In March, section of Surgery—The advantage of early surgical intervention in borderland cases, Roswell Park. Attendance, 40.

In May, section of Pathology—Dysentery and its causes, Simon Flexner, Professor of Pathology University of Pennsylvania; Notes on a few cases of smallpox from the Buffalo epidemic of 1901-1902. Stereopticon, David E. Wheeler. Attendance, 60.

The council had 7 meetings, with an average attendance of 11 2.7.

### ANNUAL REPORT OF TREASURER.

CHARLES S. JEWETT, M. D., Treasurer.

June, 17 1902.

### CURRENT FUND.

| | | |
|---|---|---|
| Balance on hand, June 11, 1901 | $263.24 | |
| Initiation fees, twenty-nine resident Fellows | 290.00 | |
| " " three non-resident Fellows | 15.00 | |
| Dues received from Fellows | 774.05 | |
| " " " estates of deceased Fellows | 25.12 | |
| Received for use of telephone | 15.18 | |
| Interest Fidelity Trust Company | 12.30 | |
| | | $1,394.89 |

### EXPENSES.

| | | |
|---|---|---|
| Printing, postage and stationery | $255.78 | |
| Janitor's services | 49.00 | |
| Collectors' fees | 18.00 | |
| Stereopticon | 20.00 | |
| Telephone | 50.28 | |
| Exchange on foreign checks | .30 | |
| Guests' expenses, medical section ($25 for last year) | 47.50 | |
| " " surgical section | 71.00 | |
| " " obstetric and gynecological section | 17.16 | |
| " " pathological section ($36 for last year) | 106.00 | |
| " " ophthalmological, laryngological and otological section (20 for last year) | 53.50 | |
| Rent | 49.00 | |
| Total expenses | $737.52 | |
| Transferred to permanent fund | 270.00 | |
| | | 1,007.52 |
| Balance on hand, June 11, 1902 | | $ 387.37 |
| To be transferred to permanent fund, July 1, 1902 | | 45.00 |
| Actual working balance | | $ 342.37 |

### PERMANENT FUND.

| | |
|---|---|
| Amount of fund, June 11, 1901 | $1,396.89 |
| Transferred from current fund | 270.00 |
| Interest Western Savings Bank | 43.56 |
| | $1,710.45 |

Increase of permanent fund in past year, 22 per cent.
Increase of permanent fund in past two years, 221 per cent.

| | |
|---|---|
| Total receipts from all sources during the year | $1,175.21 |
| Total expenses | 737.52 |
| Net increase of assets | $ 437.69 |

### SECTION OF OBSTETRICS.

W. H. MANSPERGER, M.D., Secretary.

Meetings of this section were held on the following dates:

September 24, Some experiences in obstetrical cases, Eli Long. Attendance, 28.

October 29, Concerning the diagnosis and treatment of the various forms of septic pelvic diseases, Fernand Henrotin, of Chicago. Attendance, 39.

November 26, Pelvic lesions in their relation to destructive effects upon mental disturbances, A. T. Hobbs, of London, Ont. Attendance, 32.

December 24, Concerning the management of labor cases, Stephen Y. Howell. Attendance, 15.

January 28, stated meeting of Academy, Wm. R. Pryor, New York City. Attendance, 58.

February 25, Some rare and odd cases and experiences in pelvic and abdominal surgery, C. C. Frederick; Retrodisplacements of the uterus in young girls and unmarried women—their frequency and best methods of treatment, Herman E. Hayd. Attendance, 36.

March 25, Ectopic pregnancy, H. D. Ingraham; Treatment of placenta previa, M. D. Mann. Attendance, 37.

April 22, The prophylaxsis of uterine cancer, L S. McMurtry, of Louisville. Attendance, 38.

May 27, Thoughts upon breech presentations, Dr. Van Peyma; Fibroid tumors, C. C. Frederick. Attendance, 19.

Dr. Schroeder was elected chairman; Dr. Stadlinger, secretary.

Average attendance, 33 5.9.

### SECTION OF OPHTHALMOLOGY, OTOLOGY, RHINOLOGY, AND LARYNGOLOGY.

A. G. BENNETT, M.D., Secretary.

During the season seven scientific meetings where held and one to discuss the question as to whether the section should be discontinued.

At the first meeting, September 16, a paper was presented by Swan M. Burnet, of Washington, D.C., on the question of Methyl alcohol and its effect on vision. Casey A. Wood, of Chicago, read a paper at the second meeting, October 11, on Exophthalmic Goitre. Both of these papers were scholarly, as the section had every right to expect they would be, being read by two of the foremost ophthalmologists of this country. The section regrets that these distinguished gentlemen were greeted by so small an audience and particularly that the council was only represented by one member beside the section chairman and secretary.

At the other meetings papers were presented by Drs. Cott, Hubbell, Howe, Hinkel, Grover W. Wende and Max Keiser, and interesting cases were presented by Drs. Clemesha, Blaauw, Cott, Hinkel, Flagg and Hubbell. The average attendance, 13 1.6.

At the last meeting held on Monday, May 26, it was decided to continue the section but to hold only 4 meetings during the session, and to hold these on the fifth Tuesday, which occurs every third month.

E. E. Blaauw was elected chairman; F. H. Millener, Secretary, for the ensuing year.

### SECTION OF SURGERY.

H. C. ROTH, M.D., Secretary.

The Surgical Section held eight regular meetings and entertained the Academy once. The meetings were fairly well attended, there being an average gain of 25 per cent. over the attendance of last year.

Beside having many excellent papers by our own members, the section had the pleasure of listening to papers by C. A. Hamann, of Cleveland, O.; Charles H. Frazier, Philadelphia, Pa.; Ramon Guiteras, New York; Harvey W. Cushing, Boston, Mass.; and Geo. W. Crile, Cleveland, O.

The average attendance was 27.

### SECTION OF PATHOLOGY.

IRVING PHILLIP LYON, M.D., Secretary.

During the session of 1901-1902, the Pathological Section held seven regular monthly meetings and furnished the program for one stated meeting of the Academy, making eight meetings in all, with an average attendance of 46 members, representing an average attendance of about 20 per cent. of the total membership of the Academy.

A total of eleven papers were read before the section; five by members of the Academy and six by invited guests from other cities, as follows: 1 from Ithaca, 1 from Philadelphia, 1 from New York City, 1 from Montreal, and 2 from Chicago.

Pathological specimens were exhibited by four members and by one invited guest, Dr. Loeb, of Chicago, and in addition during the year, reports of cases were made by one member and by one invited guest, Mr. Eugene Horbon, student of medicine in the University of Buffalo.

Attention is directed to the fact that a majority of the papers read were presented by distinguished guests of the Academy from various other cities in accordance with the policy adopted by the council. It is believed that this policy has stimulated interest in the meetings and its continuance is recommended by your secretary.

### SECTION ON MEDICINE.

A. E. WOEHNERT, M.D., Secretary.

During the year 1901-1902 the Medical Section held eight meetings. One meeting, that of April 8, was adjourned, there being no quorum present.

We endeavored during this year to awaken interest in the Academy by bringing in men from out of town. We met with a certain degree of success. We also set apart one night for a clinic night, inviting members to bring interesting cases. The members seemed to take kindly to the innovation, more than the average number being present. Five interesting cases were demonstrated.

The following papers were read and discussed: Intestinal dystrypsia, J. C. Hemmeter; Treatment of pneumonia, De Lancey Rochester; Dyspeptic asthma, Max Einhorn; Recurrent vomiting, A. A. Jones; Some facts and fallacies concerning tuberculin, Dr. Pryor; Diagnosis of gastric cancer, A. L. Benedict; The capillary area, Eli Long; Pneumococci and cardiac toxemia, Henry Elsner; Baby farming in Buffalo, J. Pohlman; Observation upon two interesting cases of the local manifestation of hysteria in joint and muscle, Prescott Le Breton; Jaundice, with report of cases, J. M. Anders; Heredity, Wm. C. Krauss.

Excluding the first meeting, which was a stated meeting, there were 310 members present at the seven meetings; an average of 41 3.7.

### REPORT OF TRUSTEES.

*Annual Meeting June 17, 1902.*

Since the last annual meeting of the Academy little has transpired in the way of change, save the recent rise in the world, which called us from basement to third floor. Our present quarters are in many respects preferable to those so recently abandoned. Dry, airy, commodious and easy of access through the medium of a modern electric elevator. The new rooms present an attractiveness which will be increased when certain contemplated improvements have been made.

Aside from a small stipend to be paid the elevator man, for his extra hours of labor, the expenses incurred by the Academy are not to be enhanced. The favorable conditions which have permitted a steady, healthy growth of our sinking fund are to continue, and with each passing year, the vision of a permanent ideal home for the Academy now seems less chimerical. The disposition of the various properties of the Academy is practically that of a year ago.

Our books are cared for, till such time as we may require them, by the Grosvenor Library trustees, to whom the privilege of using such as may be desired has been accorded.

In the keeping of the board of U. S. Pension Examiners, at their rooms in the new Post Office building: 1 upholstered chair; 1 long oak table; 1 short oak table; 18 hardwood arm chairs, 1 couch upholstered in leather. These are to be kept in good order and subject to demand.

The remainder of the Academy effects are at present distributed between the basement rooms just abandoned and those now occupied on the third floor. Aside from certain repairs to our chairs, the maintenance of the property during the ensuing year will apparently necessitate no drafts upon the treasury.

STEPHEN Y. HOWELL, Chairman.
J. W. GROSVENOR,
M. D. MANN.

The tellers, J. W. Grosvenor and F. W. Barrows, reported the result of election as follows: president, Herman E. Hayd; treasurer, Charles S. Jewett; trustee, Stephen Y. Howell.

The retiring president, Charles G. Stockton, made a few appropriate remarks in reference to the work of the Academy during the past year and its future, and then read his annual address, entitled, Acute gangrenous pancreatitis, traumatic and otherwise. (To be published.)

The auditing committee reported having compared the report of the treasurer with the books and vouchers and found them to be correct.

Dr. Henry R. Hopkins reported that the committee of investigation of St. Mary's Orphan Asylum and Maternity Hospital was making progress, but requested further time, and on motion more time was granted.

Dr. Julius Ullman reported a case of carcinoma of cardiac end of the stomach on which a gastrostomy was performed, and gave an exhibition of the administration of food to patient.

The newly elected president, Dr. Hayd, was duely installed into office and made a few appropriate remarks, thanking the members for having elected him to such an important office in the Academy.

It was moved and seconded that a vote of thanks be extended to the president for his masterly address. Carried.

A vote of thanks was extended to Dr. Charles S. Jewett for the successful way in which he managed the finances of the Academy; also a vote of thanks was extended to the secretary for his work during the year.

Attendance, 42. Adjourned at 10.30 p.m.

